# Evaluation of Antioxidant and Antibacterial Potentials of *Nigella sativa* L. Suspension Cultures under Elicitation

**DOI:** 10.1155/2015/708691

**Published:** 2015-08-12

**Authors:** Hera Chaudhry, Nida Fatima, Iffat Zareen Ahmad

**Affiliations:** ^1^Department of Biosciences, Integral University, Dasauli, Kursi Road, Lucknow, Uttar Pradesh 226026, India; ^2^Department of Bioengineering, Integral University, Dasauli, Kursi Road, Lucknow, Uttar Pradesh 226026, India

## Abstract

*Nigella sativa* L. (family Ranunculaceae) is an annual herb of immense medicinal properties because of its major active components (i.e., thymoquinone (TQ), thymohydroquinone (THQ), and thymol (THY)). Plant tissue culture techniques like elicitation, *Agrobacterium* mediated transformation, hairy root culture, and so on, are applied for substantial metabolite production. This study enumerates the antibacterial and antioxidant potentials of *N. sativa* epicotyl suspension cultures under biotic and abiotic elicitation along with concentration optimization of the elicitors for enhanced TQ and THY production. Cultures under different concentrations of pectin and manganese chloride (MnCl_2_) elicitation (i.e., 5 mg/L, 10 mg/L, and 15 mg/L) showed that the control, MnCl_2_ 10 mg/L, and pectin 15 mg/L suspension extracts greatly inhibited the growth of *E. coli*, *S. typhimurium*, and *S. aureus* (MIC against *E. coli*, i.e., 2.35 ± 0.8, 2.4 ± 0.2, and 2.46 ± 0.5, resp.). Elicitation decreased SOD enzyme activity whereas CAT enzyme activity increased remarkably under MnCl_2_ elicitation. MnCl_2_ 10 mg/L and pectin 15 mg/L elicitation enhanced the DPPH radical inhibition ability, but ferric scavenging activity was comparable to the control. TQ and THY were quantified by LC-MS/MS in the cultures with high bioactive properties revealing maximum content under MnCl_2_ 10 mg/L elicitation. Therefore, MnCl_2_ elicitation can be undertaken on large scale for sustainable metabolite production.

## 1. Introduction

The use of spices as food additives, flavouring agents, and also digestive stimulant tonic has been practiced widely since ancient times. These attributes, largely empirical, nevertheless efficacious, have earned them pharmacological applications in the indigenous systems of medicine. Among various medicinal spices* Nigella sativa* L. (family Ranunculaceae) has emerged as a miraculous herb with a wide spectrum of pharmacological activities.* N. sativa* seeds are most extensively studied, both phytochemically and pharmacologically. Both seeds and oils are known to possess various health properties like antitumour activity [1], antioxidant activity [2], anti-inflammatory activity [3], antibacterial activity [4], and a stimulatory effect on the immune system [5] because of which they are often used as nutritional supplement. Also it is reported that different crude extracts of* N. sativa* have shown effectiveness against multiantibiotic resistance bacterial isolates [6]. Black seed extracts have also proved to be potent antimicrobial agents against certain pathogenic Gram positive and Gram negative bacteria [7]. Further, it is known that its oil and fraction act as potent* in vitro* free radical scavengers which are correlated well with its total content of polyunsaturated fatty acids, unsaponifiables, phospholipids, and phytoconstituents as well as the initial peroxide values of crude oil [8]. These strong antimicrobial and antioxidant properties of different extracts and oil of* N. sativa* are due to the rich presence of chemically varied bioactive compounds.

Techniques of plant tissue culture have proved to be the best alternatives in supplementing traditional agriculture for the industrial production of medicinal plant derived metabolites [9]. Among the various techniques, elicitation has proved to be an effective strategy for the enhanced production of commercially and medicinally important bioactive compounds. Elicitors are stress agents that induce the accumulation of secondary metabolites along with phytoalexins in plants, as well as in plant cultures, and the phenomenon is known as elicitation [10]. Elicitors can be both biotic (pectin, chitin, cellulose, glycerol, jasmonic acid, etc.) and abiotic (heavy metals, salts, herbicides, pH, temperature, etc.) depending upon their nature and source. Plant cells recognize the elicitors by interacting with them via specific receptors present on the plasma membranes. The elicitor-receptor interactions generate many downstream signals which then activate nuclear plant defense genes such as phytoalexins. The action of local, systemic signal molecules and putative plasma membrane receptors is solely responsible for the initiation of the pathway. Many heavy metals (Ag, V, Cr, Ni, Pb, Hg, Cu, and Mn) and their salts are reported to have enhanced the metabolite production like copper sulphate facilitating the accumulation of cardiac glycosides up to 26 times in cell cultures of* Digitalis lanata* [11]. Furthermore, biotic elicitation, for example, pectin treatment, has also enhanced the induction of oleanolic acid in cell suspension cultures of* Calendula officinalis* [12]. The accumulation of secondary products is regarded as a part of the defense system of the intact plant.

The present study was conducted to demonstrate the antioxidant and antibacterial efficacy against different Gram positive and Gram negative bacteria of the* N. sativa* epicotyl suspension cultures under the effect of different concentrations of biotic (pectin) and abiotic elicitor (MnCl_2_). Further, the effect of elicitation on cell growth, thymoquinone (TQ) and thymol (THY) production from the cell suspension cultures of* N. sativa* was examined.

## 2. Materials and Methods

### 2.1. Chemicals

Murashige and Skoog (MS) medium, kinetin, naphthalene acetic acid, pectin, MnCl_2_, methanol, toluene, chloroform, ethanol, diethyl ether, benzene, streptomycin, ciprofloxacin, doxycycline, ampicillin, ofloxacin, dimethyl sulfoxide, DPPH (1,1-diphenyl-2-picrylhydrazyl), sodium acetate, glacial acetic acid, hydrochloric acid, TPTZ (2,4,6-tri[2-pyridyl]-s-triazine), ferrous sulphate, ferric chloride, nitro blue tetrazolium, ethylenediaminetetraacetic acid (EDTA), riboflavin, hydrogen peroxide, methionine, dipotassium phosphate, monopotassium phosphate, and thymol (THY) were purchased from HIMEDIA; thymoquinone (TQ) was purchased from Sigma Aldrich.

### 2.2. Plant Material

Seeds of* N. sativa* were procured from a local grocery store in Lucknow, India, and authenticated at the National Botanical Research Institute, Lucknow. Seeds were surface-sterilized by washing thoroughly under tap water containing few drops of Tween-20 and then rinsed with 70% ethanol for 30 seconds followed by washing with sterile water twice. They were then immersed in 0.2% mercuric chloride solution for 5 min after which they were rinsed with sterile water twice, were allowed to grow in glass petri plates having threefold of damp blotting paper in distilled water at 25 ± 2°C under aseptic condition in dark for three days till sprouting, and, thereafter, were exposed to light (photoperiod of 14/10 h, 100 *μ*mol m^−2^ s^−1^), where complete germination with leaf, epicotyl, hypocotyl, and root took eleven days [13].

### 2.3. Callus Induction and Establishment of Suspension Cultures

Explants leaves (40–50) and segments (0.5 cm) of epicotyl, hypocotyl, and root from the 11th day seedling were surface-sterilized and cultured in triplicate on solidified Murashige and Skoog (MS) medium supplemented with kinetin (Kn) 2 mg/L + naphthalene acetic acid (NAA) 1 mg/L [14]. Cultures were kept under a photoperiod of 16/8 h white fluorescent light at 25 + 2°C for a month. Cell suspension cultures were derived from friable epicotyl callus in Erlenmeyer flasks (250 mL) containing 100 mL of liquid MS medium supplemented with Kn 2 mg/L + NAA 1 mg/L in triplicate. Cultures were maintained for a month at 110 rpm (Remi Orbital Shaker Incubator, Model number IHC-2410) at 25 ± 2°C, 3000 lux, and 16/8 h photoperiod and growth was determined by loss of weight by dissimilation [14].

### 2.4. Effect of Biotic and Abiotic Stress on Growth, Biomass Accumulation, and Metabolite Production

A cell biomass of 50–75 mg from the stock culture was transferred into Erlenmeyer flasks of separate groups, containing 100 mL medium with Kn 2 mg/L + NAA 1 mg/L in triplicate under three different concentrations of pectin and MnCl_2_, that is, 5 mg/L, 10 mg/L, and 15 mg/L (as they have given the best results in enhancing metabolite production in the epicotyl callus of* N. sativa* in our previous study) [15]. pH of the medium was adjusted to 5.8 before autoclaving. Cultures were maintained at 110 rpm, 16/8 h, and 25 ± 2°C for a month and growth was determined by loss of weight by dissimilation, TQ and THY production was estimated by TLC and LC-MS/MS, and growth index was calculated by the following equation:

(1) Growth index=final cell weight − initial cell weightinitial cell weight.

### 2.5. Estimation of Thymol and Thymoquinone by TLC

#### 2.5.1. Extraction Method

Cells from different suspension cultures were ground in 100% of methanol using pestle and mortar at room temperature with periodical mixing on shaker overnight, filtrated using Whatman Grade number 1 filter paper, medium particle retention (pore size 11 *µ*m), and concentrated at 45°C up to 5 mL. Extracts were stored at −20°C for further use.

#### 2.5.2. TLC for Thymol

Thymol presence was determined by using Wagner and Bladt [16] method with slight modification and *R*_*f*_ values of the spots were measured.

#### 2.5.3. TLC for Thymoquinone

Thymoquinone presence was determined by using Suthar et al.'s [17] method with slight modification and *R*_*f*_ values of the spots were measured.

### 2.6. DPPH Radical Scavenging Activity

The free radical inhibition activity of different extracts was determined by 1,1-diphenyl-2-picrylhydrazyl (DPPH) [18]. Briefly, different concentrations of crude extract and 0.1 mM methanolic DPPH solution were added to 3 mL of reaction mixture, incubated for 30 minutes. Absorbance was taken at 517 nm lower absorbance of the reaction mixture indicating higher free radical inhibition activity. All extracts were analyzed in triplicate. Ascorbic acid was taken as a standard:

(2) $$\begin{array}{c} \text{DPPH inhibition activity }\left(\;\%\;\right)=\left(\;\frac{A0-A1}{A0}\;\right)\times 100, \end{array}$$

where *A*1 is the absorbance of the sample and *A*0 is the absorbance of the control, respectively.

### 2.7. Ferric Reducing Antioxidant Power (FRAP) Assay

The FRAP assay measures the change in absorbance at 593 nm owing to the formation of a blue colored ferrous; Fe^2+^-tripyridyltriazine compound from the colorless oxidized ferric Fe^3+^ forms by the action of electron donating antioxidants [19]. The FRAP reagent consisted of 300 mM acetate buffer, 10 mM TPTZ (2,4,6-tripyridyltriazine) in 40 mM HCl, and 20 mM FeCl_3_·6H_2_O in the ratio of 10 : 1 : 1. For the standard curve FeSO_4_ solution dilution from 0.2 mM to 1.6 mM concentration was prepared from the stock and processed in similar way. Results of percentage scavenging were compared with those of BHT, ascorbic acid, quercetin, and catechin:

(3) $$\begin{array}{c} \text{FRAP scavenging activity }\left(\;\%\;\right)=\left(\;\frac{A0-A1}{A0}\;\right)\times 100. \end{array}$$

### 2.8. Assay of Antioxidant Enzyme Activities

#### 2.8.1. Enzyme Extraction

The samples were prepared as described by Mukherjee and Choudhuri [20] with some modifications. Samples were finely ground; 10 mL of 100 mM phosphate buffer (KH_2_PO_4_/K_2_HPO_4_) pH 7.0, containing 0.1 mM Na_2_EDTA and 0.1 g of polyvinylpyrrolidone (PVP), was added to them. The homogenate was filtered through Whatman filter and centrifuged at 15000 ×g for 10 min at 4°C; supernatant was recentrifuged at 18000 ×g for 10 min; the supernatant was stored at 4°C for enzyme assay.

#### 2.8.2. Superoxide Dismutase Activity

SOD activity was measured in a reaction solution (3 mL) according to the method of Giannopolitis and Ries [21]. SOD activity was determined by measurement of inhibition of photochemical reduction of nitro blue tetrazolium (NBT) at 560 nm, where one unit of SOD activity was defined as the amount of enzyme causing 50% inhibition of photochemical reduction of NBT.

#### 2.8.3. Catalase Activity

CAT activity was assayed by the method of Aebi [22] in a reaction solution (3 mL). The activity of catalase was estimated by the decrease of absorbance at 240 nm for 1 min as a consequence of H_2_O_2_ consumed [23].

### 2.9. Determination of* In Vitro* Antimicrobial Effect

#### 2.9.1. Pathogenic Bacterial Strains Used for the Study

Pure cultures of five test pathogenic bacteria isolates, namely,* Escherichia coli* (NCIM 2065),* Staphylococcus aureus* (NCIM 2099),* Klebsiella pneumoniae* (NCIM 2957),* Salmonella typhimurium* (NCIM 2501), and* Bacillus cereus* (NCIM 2156), were procured from NCIM, Pune, India.

#### 2.9.2. Inoculum Preparation

Active cultures for each bacterial species were prepared by transferring a loopful of cells from the stock cultures to nutrient broth, incubated without agitation for 24 hrs at 37°C; further the cultures were diluted with fresh nutrient broth to achieve optical densities corresponding to 10^6^ cfu mL^−1^ [24].

#### 2.9.3. Broth Dilution Assay

Modified broth dilution technique was used to determine the minimum inhibitory concentration (MIC) values. Log phase cultures of bacteria were diluted 100-fold in NB (100 *μ*L bacterial cultures in 10 mL NB which contained 10^5^ cfu of bacteria). Gradually increasing concentrations of the extracts were added to test tubes containing the bacterial cultures. The tubes were incubated at 37°C for 18–24 hours. Visible turbidity and optical density of cultures were determined at 620 nm using NB as control to elucidate MIC [25].

#### 2.9.4. Agar Well Diffusion Assay

Agar well diffusion method was used to test the antibacterial effect of different suspension culture crude extracts [26, 27]. Media plates (11 cm in diameter) were prepared with nutrient agar. A total of four wells (7 mm in diameter) per agar plate were cut. For test, three doses of extract (25, 50, and 75 *μ*L/well) were used. Standard antibiotics, streptomycin (30 *μ*g), ciprofloxacin (10 *μ*g), doxycycline (30 *μ*g), ampicillin (10 *μ*g), and ofloxacin (5 *μ*g), were used as positive control and dimethyl sulfoxide (75 *μ*L/well) was used as negative control. A 100 *μ*L (10^5^ cfu) of diluted microbial suspension was swabbed on nutrient agar plates. Extracts and positive control were added separately to each well and allowed to diffuse at room temperature for 15–20 min. Plates were incubated at 37°C for 24 h after which they were examined for zones of growth inhibition and the diameter of these zones was measured. The assay was repeated three times for each extract. The antimicrobial effects were recorded as the mean diameter of the resulting inhibition zones of growth in millimeter.

### 2.10. Estimation of Thymol and Thymoquinone by LC-MS/MS

Extraction from different suspension cultures was done in 100% of HPLC grade methanol using mortar and pestle with periodical mixing on shaker overnight and centrifuged at 5000 ×g for 10 min, and then the supernatant (5 mL) was collected for analysis.

Analysis was performed using Triple Quadrupole LC-MS/MS Mass Spectrometer (MS-Manufacturer—AB Sciex Instruments, Model 1034067 V, Serial number V210201201) turbo spray interface. The separation was carried out in Acquity UPLC BEH apparatus with an autosampler equipped with C18 column dimension—2.1 × 50 mm. Column temperature was kept ambient, mobile phase methanol and water in ratio of 90 : 10, source temperature 200.0°C at set point, injection volume 10.0 *µ*L, and run time 2 min. All acquisitions were performed under positive ionization mode with a capillary voltage of 3500 V. Data acquisition and processing were done using the software version Analyst 1.6.

Q_1_MS total chromatogram was run at positive mode and the nebulizer gas (GS1, 10.00), heater gas (GS2, 10.00), temperature (TEM, 200.00), declustering potential (DP), entrance potential (EP, 10.00), collision energy (CE), and collision exit potential (CXP, 9.00) of the compounds were optimized. CXP gave the daughter ion of the parent ion. Multiple Reaction Monitoring (MRM) of the compound's daughter ion was developed. The chromatogram developed gave separate peaks of different area for two compounds which was used to quantify the two compounds in the samples against the standard thymol and thymoquinone in terms of *μ*g/gm. Best peak of thymoquinone was developed at 151.100/109.100 (DP—start 52.00; stop 52, CE—start 17.00; stop 17.00) and best peak of thymol was developed 165.100/137.100 (DP—start 70.00; stop 70.00, CE—start 35.00; stop 35.00).

### 2.11. Statistical Analysis

Data were statistically analyzed and the results were expressed as means (±SD) of average of three replicates (*n* = 3). *p* values (≤0.05) were considered as significant compared to the respective controls.

## 3. Results and Discussion

### 3.1. Callusing Response and Establishment of Suspension Cultures

Callusing results showed that, among the different explants (leaf, epicotyl, hypocotyl, and root), epicotyl segments (0.5 cm long) gave the best and fast callusing response with creamish white friable callus. The stock suspension cultures were initiated by these epicotyl calluses which were subcultured every week. A sigmoidal growth curve was obtained with all five growth phases, that is, lag, exponential, linear, stationary, and progressive decline. Maximum growth and longest stationary phase were achieved in suspension culture supplemented with Kn (2 mg/L) + NAA (1 mg/L) combination.

Plant tissue culture techniques are of immense importance for the production of myriad of useful secondary metabolites as compared to the whole plant or microbial system [28, 29]. In the present study, an epicotyl suspension culture was established and maintained under suitable conditions of aeration, agitation, light, temperature, and other physical parameters [30].

### 3.2. Effect of Different Concentrations of Pectin and MnCl_2_ on Growth

*N. sativa* epicotyl suspension cultures were subjected to three different concentrations of biotic and abiotic elicitors. Growth pattern under elicitation was determined, where cultures under pectin and MnCl_2_ reported early cell death which was due to the deleterious effect of elicitation. Maximum growth inhibition caused by both the elicitors, that is, pectin and MnCl_2_, was recorded in the cultures under 15 mg/L of elicitation having growth index of 2.533 and 2.403, respectively (Table 1). In contrast to this control cultures gave maximum growth index of 3.413 followed by 5 mg/L and 10 mg/L of pectin and MnCl_2_ elicitation where it was 2.850, 2.786 and 2.906, 2.500, respectively. The abiotic elicitation thus proved to be more deleterious than the biotic elicitation towards cell division, fresh weight accumulation, and growth index and, therefore, showed a dose-dependent growth inhibition.

Pectin elicitation used in the present study clearly supports the facts of the previous findings that elicitation facilitates growth and enhances metabolite production. Pectin elicitation showed a dose-dependent effect on the cell growth and biomass accumulation of the* N. sativa* epicotyl suspension cultures as seen in the cultures of* Mucuna pruriens* in a study conducted by Raghavendra et al. [31]. Further, results of our study regarding the effect of abiotic elicitor (MnCl_2_) on culture growth are in accordance with the results of Ghorpade et al. [32] conducted on* Boswellia serrata* callus cultures which showed a deleterious effect of increasing concentration of the abiotic elicitors on the biomass accumulation.

### 3.3. TLC Analysis for TQ and THY under Different Concentrations of Pectin and MnCl_2_

The pectin elicited cultures were preliminarily demonstrated for production of TQ and THY by TLC. The accumulation of TQ and THY showed dose-dependent response as in the case of both the metabolites band intensity and color increased with the increasing concentration of the elicitor (Table 2). Bands of *R*_*f*_ values 0.76 and 0.82 similar to the standards used for THY and TQ, respectively, were recorded in all three cultures with a more prominent band in cultures under 15 mg/L elicitation though of less intensity as compared to the control cultures showing clearly that higher amount of pectin elicitation facilitated the production of TQ and THY. Bands of other metabolites of *R*_*f*_ values, that is, 0.97, 0.73, 0.60, and 0.43, were also recorded in the THY TLC. The TQ TLC results also showed the presence of two more bands of *R*_*f*_ values 0.95 and 0.68.

The TLC results under MnCl_2_ elicitation reported the production of THY along with four other metabolites with *R*_*f*_ values 0.76, 0.97, 0.73, 0.60, and 0.43, respectively (Table 2). A diminished pink color band of *R*_*f*_ value 0.76 similar to the standard THY used was seen in the cultures under 5 mg/L of elicitation; on the other hand culture under 10 mg/L showed a prominent band somewhat similar to the control culture. The elicitation of 15 mg/L totally inhibited the THY production. TLC results of TQ production also followed a similar pattern as in THY. Culture under 5 mg/L elicitation gave a diminished yellow color spot of less intensity as compared to the control culture of *R*_*f*_ value 0.82 along with two other spots of *R*_*f*_ values 0.95 and 0.68. The intensity of TQ spot in the culture under 10 mg/L of the MnCl_2_ elicitation was similar to the control culture and also reported the presence of other two spots recorded in 5 mg/L but of more intensity. Elicitation of MnCl_2_ 15 mg/L totally inhibited the occurrence of TQ as no spot of TQ was reported, thus reporting serious harmful effect of elevated concentrations of abiotic elicitors on TQ and THY production.

Elicitors are known to cease the* in vitro* culture growth temporarily or permanently. This cessation may also lead to a defense response by switching from primary metabolism to secondary one [33]. TLC results and high concentrations quantified in LC-MS/MS results for both the metabolites in 15 mg/L of pectin elicitation are justified by the above statement where higher concentration leads to the conversion of the primary metabolites to secondary ones in order to prevent cell death. Earlier reports have attempted to correlate changes of HMGR enzyme activity with the induced synthesis of particular isoprenoids as this is a key enzyme in the terpenoid biosynthesis. Suzuki et al. [34] correlated a transient induction of HMGR activity with the accumulation of ipomeamarone, a furanosesquiterpenoid, in sweet potato tissue infected with* Ceratocystis fimbriata*. Stermer and Bostock [35] have shown a transient induction of HMGR activity in potato discs stimulated to produce sesquiterpenoids by application of arachidonic acid. Therefore, MnCl_2_ elicitation somehow triggered the TQ and THY production by inducing the activity of HMGR though no such evidence is still available. Further, the study of Moses and Mukundan [36] on THY enhancement by lower concentration MnCl_2_ did not exhibit noticeable increase in THY accumulation; in contrast to this our results showed that even lower dose of MnCl_2_, that is, 10 mg/L, elicited the metabolite production.

### 3.4. Antioxidant Capacities of Different Cultures under Biotic Abiotic Elicitation

#### 3.4.1. DPPH Free Radical Inhibition Activity

**Figure 1:**
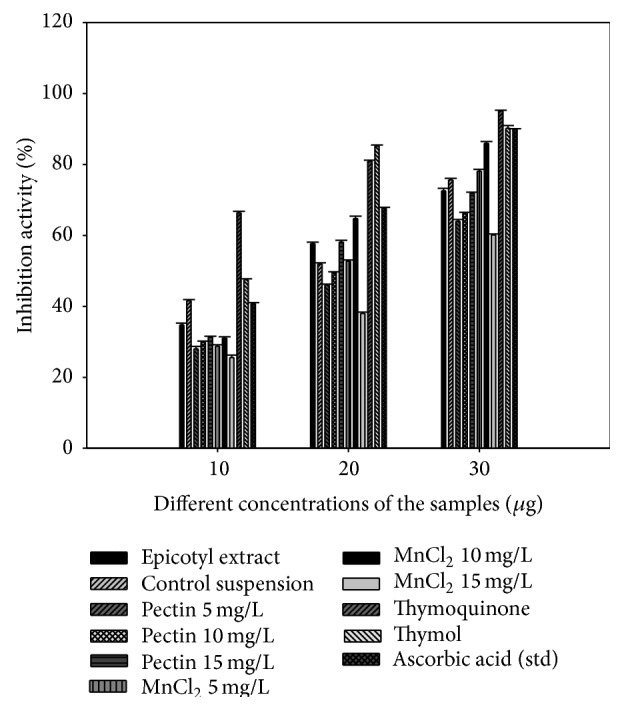
Percentage inhibition activity of different suspension cultures under elicitation.

DPPH radical inhibition activity of various tissue culture generated extract is depicted in Figure 1. The inhibition activity recorded for the cultures under pectin elicitation increased with the increasing elicitor concentration and pectin 15 mg/L elicitation recorded maximum inhibition comparable to the cultures without elicitation (control suspension), epicotyl explant extract, and to some extent the standard, that is, ascorbic acid over the 18.62 IC_50_ value (Table 3). In the case of MnCl_2_ elicitation 5 mg/L and 10 mg/L elicitation inhibited DPPH radical at higher level than the pectin elicited cultures, but maximum inhibition was shown by cultures under 10 mg/L elicitation which was higher than the epicotyl explant extract and cultures without elicitation (control cultures) also comparable to the standard ascorbic acid used over a value of 16.44 IC_50_ (Table 3). The inhibition caused by cultures under biotic and abiotic elicitation was far less than the pure TQ used but very near to THY. Standards and all the extracts showed a dose-dependent inhibition on the DPPH radicals.

In the present work, epicotyl suspension cultures under less favourable conditions (biotic and abiotic elicitation) showed increased levels of antioxidant metabolites, that is, TQ and THY, which contributed to reducing the stress generated in these circumstances. The observed high levels of DPPH radical scavenging capacity in the MnCl_2_ elicited cultures were comparable to THY and ascorbic acid could reflect the expression of antioxidant biosynthetic pathways resulting in prevention or minimization of the cytotoxic impact of ROS formed during stress conditions, as described against chilling [37] and salt stress [38]. The previous studies done on the antioxidant activity of* N. sativa* seeds were in the range of IC_50_ 2.26–28.8 mg/mL [2, 39]. The values of both IC_50_ and EC_50_ obtained in this study were lower than those of previous studies, thereby indicating a higher antioxidant activity in the suspension samples. The results of this study are also in accordance with the previous study that the higher enzyme activity and antioxidant properties are attributed to increased TQ and THY production in them under elicitation which are known antioxidants.

#### 3.4.2. FRAP-Ferric Ion Scavenging Activity

**Figure 2:**
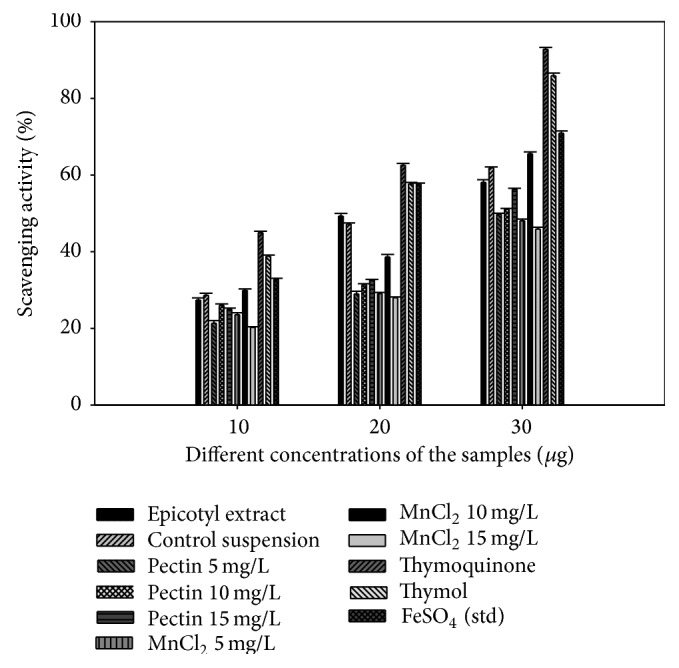
Percentage scavenging activity of different suspension cultures under elicitation.

Results of Fe (III) reduction demonstrated that all the tissue culture generated extracts had lower reducing ability than the radical scavenging activity (Figure 2). Pectin elicited cultures followed the similar scavenging pattern as in DPPH with pectin 15 mg/L being the potent scavenger at an EC_50_ value of 27.8, whereas MnCl_2_ 10 mg/L elicited cultures showed higher reducing ability than the pectin elicitation, epicotyl explant extract, and the cultures without elicitation (control suspension culture) at an EC_50_ value of 23.46 which was comparable to the standard used, that is, FeSO_4_ (Table 3). The chelating effect of all the extracts increased with an increase in their concentrations which may be due to the increase in the amount of the secondary metabolites present in the extracts.

The protective effects of plant are ascribed because of their several components; correspondingly the metabolites like thymoquinone, carvacrol, thymol, cymene, t-anethole, and 4-terpineol confer major antioxidant properties to* N. sativa* [40, 41]. Results of the study clearly have stated the positive effect of both elicitors towards TQ and THY production which provided the cultures with remarkable scavenging activity.

### 3.5. Antioxidant Enzymes Activity under Biotic and Abiotic Elicitation

#### 3.5.1. Superoxide Dismutase

**Figure 3:**
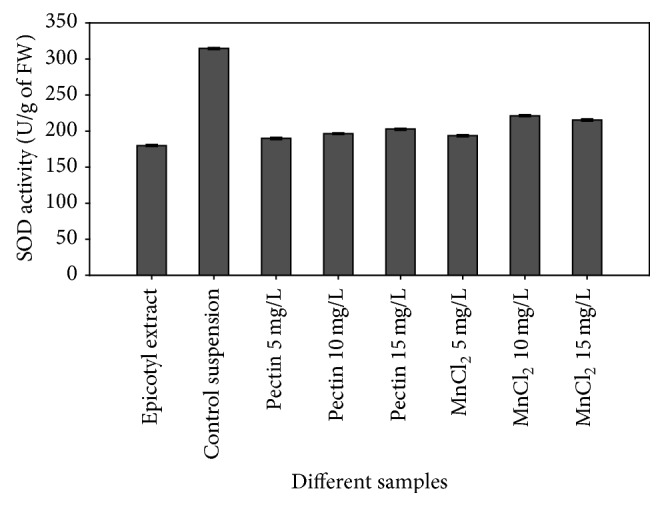
Activity of superoxide dismutase enzyme in terms of U/gm of fresh weight of samples.

Results showed that both biotic and abiotic elicitors had an effect on the activity of antioxidant enzyme SOD. As the concentration of elicitors was increased the activity of the enzyme was also enhanced. MnCl_2_ (10 mg/L) proved to be the best inducer (Figure 3) with respect to SOD activity as compared to pectin. On the contrary, SOD enzyme activity showed a remarkable decrease when compared to the cultures without elicitation (control). This might be due to the excessive consumption of the enzyme in combating the increased ROS level under elicitation. The change in the activity of SOD under elicitation was too prominent; therefore, it can be said that its presence in all samples suggests that this enzyme may participate in protecting suspension cells against free superoxide radicals.

There are many reports in support of the increased activities of SOD under abiotic stresses induced with tissue culture techniques in a wide range of plant species, including heavy metals, such as Al, Cd, Mn, Cr, and Cu, salt, and drought [42–48]. Therefore, the increased level of SOD activity under MnCl_2_ elicited suspension cultures in comparison to the pectin elicitation is in justification of the previous studies.

#### 3.5.2. Catalase

**Figure 4:**
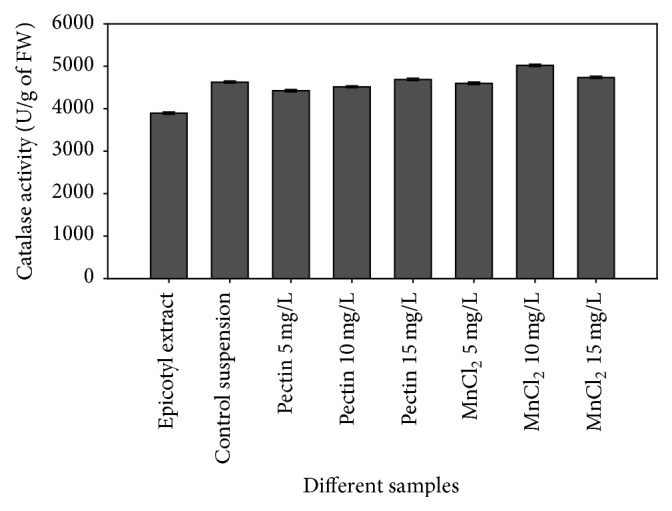
Activity of catalase enzyme in terms of U/gm of fresh weight of samples.

In the activity of CAT enzyme a significant increase was observed in the elicited cultures when compared to the epicotyl explant extract and cultures without elicitation (control suspension culture). Pectin 10 mg/L and MnCl_2_ 5 mg/L reported almost similar level of enzyme activity, whereas pectin 15 mg/L, MnCl_2_ 10 mg/L, and MnCl_2_ 15 mg/L reported a higher enzyme activity (Figure 4). The considerable increase seen in the catalase enzyme activity particularly under MnCl_2_ elicitation might have aroused so as to neutralize the large amount of generated H_2_O_2_ in the elicited cultures as the end product of SOD catalyzed reaction when compared to control cultures.

Plant products are rich sources of phytochemicals as is the extract of this study and have been found to possess a variety of biological activities including antioxidant, cytotoxic, and hepatoprotective potentials. They are excellent reducing agents and reverse oxidation by donating electrons and/or hydrogen ions [49]. Increased CAT enzyme activities reported in the suspension cultures under abiotic elicitation in this study are supported by the facts of previous findings which have demonstrated that CAT activities are also induced under abiotic stresses in different* in vitro* conditions in different plants, including heavy metals, such as Al, Cd, Mn, Cr, and Cu, salt, and drought [42–48].

### 3.6. *In Vitro* Antimicrobial Effect of Different Suspension Extracts

**Figure 5:**
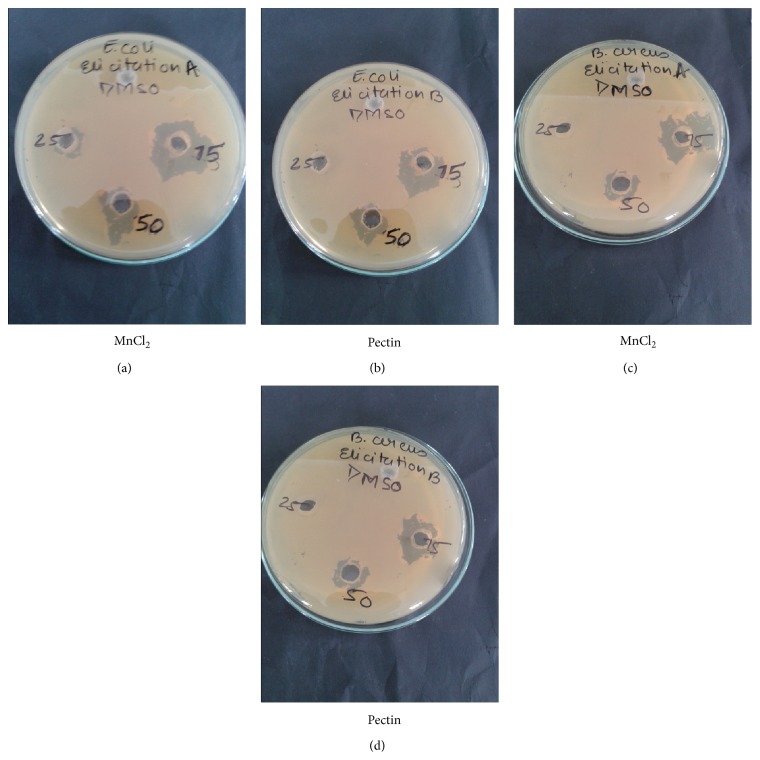
Zones of inhibition shown by different suspension culture extracts of* N. sativa* against* E. coli* and* B. cereus*.

The different methanol extracts of epicotyl and suspension cultures grown under biotic and abiotic elicitors were studied for their antibacterial potential against five Gram positive and Gram negative pathogenic bacterial strains, results of which indicated that different extracts showed different degrees of growth inhibition higher than the standard drugs used depending on the quantity of the metabolites quantified, type of elicitation, dose, and bacterial strains (Table 5). Among the five tested bacterial strains, almost all the extracts showed a maximum degree of inhibition with lowest minimum inhibitory concentrations (MIC) towards* E. coli* and* B. cereus*, that is, inhibiting the growth of both Gram positive and Gram negative bacteria (Figure 5).

Among the biotic and abiotic elicitation, control cultures, and the* in vitro* germinated epicotyl extract, control suspension culture reported highest activity against* E. coli* (32 ± 0.4) with MIC value 2.35 ± 0.8 *µ*g mL^−1^ at lowest dose of 25 *µ*L (25 mg sample) (Table 4). Likewise, in the case of cultures grown under elicitation of pectin 15 mg/L and MnCl_2_ 10 mg/L, highest zone of inhibition was recorded against* B. cereus* (30 ± 0.7) and* E. coli* (30 ± 0.2) (Table 5) with MIC values 2.55 ± 0.6 and 2.46 ± 0.2 *µ*g mL^−1^ (Table 4), respectively, at lowest dose of 25 *µ*L (25 mg sample). Further, MnCl_2_ 15 mg/L elicited culture recorded least inhibitory activity followed by epicotyl, with maximum inhibitions against* E. coli* 18 ± 0.2, MIC 4.15 ± 0.3 *µ*g mL^−1^; 22 ± 0.3, MIC 3.61 ± 0.5, respectively. Results of the* in vitro* generated extracts were far better when compared with standard antibiotics used as positive control.

*N. sativa* is known to have very strong antibacterial background mainly due to lethal effects of its active components, that is, TQ and THY, towards the microbial growth [50–52]. Antibacterial results of our study are in accordance with the results of Islam et al. [25] and Kamal and Ahmad [53], as the methanolic extracts of different elicited cultures proved to be potent natural inhibitors. The inhibitory activity of plant or tissue culture extracts depends upon the type of explant used, concentration, and microbes tested [54]. Findings of the present study are an extension of our previous work where MnCl_2_, CoCl_2_, cellulose, and pectin elicited epicotyl callus extracts showed remarkable antibacterial properties [15]. Therefore, this may be a reason for the variation in the inhibitory activity of different extracts of* N. sativa* against the bacterial strains where maximum inhibition was marked by MnCl_2_ 10 mg/L elicited epicotyl followed by pectin 15 mg/L elicited culture extracts.

### 3.7. Estimation of Thymol and Thymoquinone by LC-MS/MS

**Figure 6:**
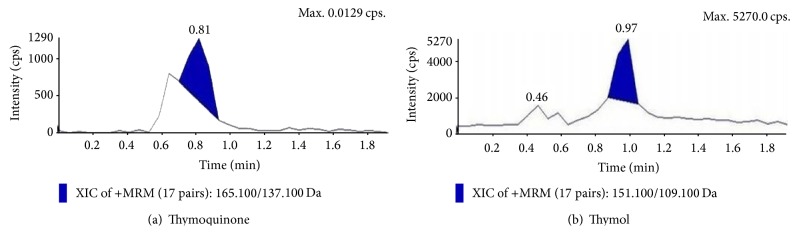
Extracted ion chromatograms and mass spectra of* in vitro* grown epicotyl extracts of* N. sativa*.

**Figure 7:**
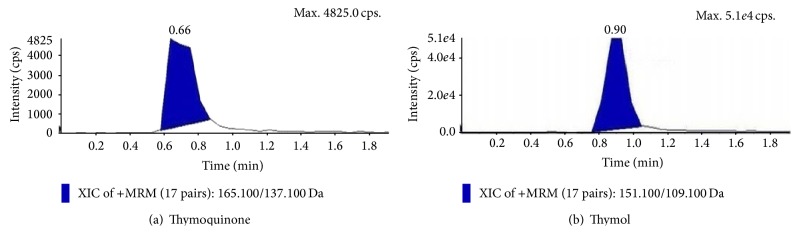
Extracted ion chromatograms and mass spectra of epicotyl derived suspension cultures (control cultures) of* N. sativa*.

**Figure 8:**
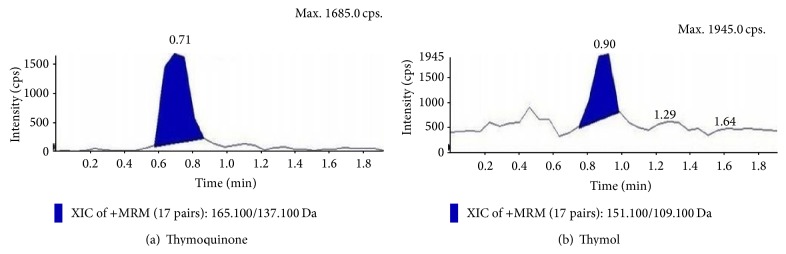
Extracted ion chromatograms and mass spectra of epicotyl derived suspension cultures (MnCl_2_ 10 mg/L elicitation) of* N. sativa*.

**Figure 9:**
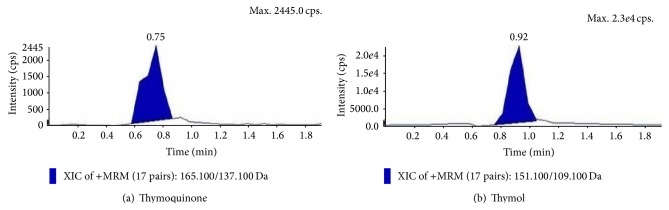
Extracted ion chromatograms and mass spectra of epicotyl derived suspension cultures (pectin 15 mg/L elicitation) of* N. sativa*.

Suspension cultures which marked the prominent presence of thymoquinone and thymol in the TLC analysis and also proved to be potent inhibitors towards the pathogenic bacterial isolates and different reactive free radicals were further quantified for the presence of TQ and THY in them. Quantitative analysis of the epicotyl explant showed a total content of TQ 1.843 ± 0.34 *µ*g/gm and THY 1.08 ± 0.27 *µ*g/gm (Figures 6(a) and 6(b)), whereas in control suspension cultures, it was TQ 2.82 ± 0.41 *µ*g/gm and THY 1.99 ± 0.21 *µ*g/gm (Figures 7(a) and 7(b)). In contrast to this MnCl_2_ 10 mg/L elicitation marked the highest presence of the metabolites, that is, TQ 2.90 ± 0.33 *µ*g/gm and THY 1.86 ± 0.29 *µ*g/gm, showing the positive effect of abiotic elicitation followed by pectin elicitation of 15 mg/L TQ 2.64 ± 0.27 *µ*g/gm and THY 1.72 ± 0.5 *µ*g/gm (Figures 7(a), 7(b), 8(a), 8(b), 9(a), and 9(b)).

The higher degree of antioxidant and antibacterial activities demonstrated by the suspension extracts was in regard to the enhanced production of the two very important metabolites, that is, TQ and THY, along with other terpenoids which is comparable to the quantification results of TQ in oil and extracts cited by Iqbal et al. and Solati et al. [55, 56].

## 4. Conclusion

The present study was carried out to exploit the potential of* N. sativa* epicotyl suspension culture for thymoquinone and thymol production and their efficacy as potent antioxidants and antibacterial agents. Therefore, this is a first report on response of growth and metabolite production in epicotyl suspension cultures of* N. sativa* under the effect of biotic and abiotic elicitation. Results showed that MnCl_2_ elicitation enhanced the production of thymoquinone and thymol; further, these extracts demonstrated strong antioxidant and antibacterial properties against Gram positive and Gram negative bacteria due to increased phytochemical accumulation. Henceforth, it could be concluded that these elite cell lines can be used for sustainable production of thymoquinone and thymol which will be helpful in understanding the biosynthetic mechanism of these metabolites under* in vitro* conditions.

## Figures and Tables

**Table 1:** Effect of different concentrations of pectin and MnCl_2_ elicitation on growth index.

S. number	Different concentrations of elicitors	Growth index
1	Control	3.413
2	Pectin 5 mg/litre	2.850
3	Pectin 10 mg/litre	2.786
4	Pectin 15 mg/litre	2.533
5	MnCl_2_ 5 mg/litre	2.906
6	MnCl_2_ 10 mg/litre	2.500
7	MnCl_2_ 15 mg/litre	2.403

**Table 2:** TLC screening of thymoquinone and thymol production under different concentrations of pectin and MnCl_2_ elicitation.

S. number	Color of spot	*R*_*f*_ value	Different concentrations of pectin and MnCl_2_							
			A	B	C	D	E	F	G	H
TLC for thymol										
1	Thymol std. pink	0.76	+	+++	+	+	++	+	+++	−
2	Purple	0.97	−	+	−	+	+	+	+	+
3	Light blue	0.73	+	++	++	+	+	+	+	−
4	Light blue	0.60	++	++	−	+	++	−	++	−
5	Pinkish purple	0.43	+	+	+	+	−	+	−	−

TLC for thymoquinone										
1	Thymoquinone std. yellow	0.82	++	+++	+	+	++	+	+++	−
2	Brownish yellow	0.95	+	+	+	+	−	+	+	+
3	Brownish yellow	0.68	+	++	−	+	+	++	+	+

A = epicotyl extract; B = control suspension culture; C = pectin 5 mg/L elicitation; D = pectin 10 mg/L elicitation; E = pectin 15 mg/L elicitation; F = MnCl_2_ 5 mg/L elicitation; G = MnCl_2_ 10 mg/L elicitation; H = MnCl_2_ 15 mg/L elicitation; “−” = absent; and “+” = present.

**Table 3:** Antioxidant activity of the different suspension extracts in terms of IC_50_ and EC_50_ (*µ*g/mL).

S. number	Different tissue culture generated extracts	DPPH IC_50_ (*µ*g/mL)	FRAP EC_50_ (*µ*g/mL)
1	Epicotyl extract	17.62	24.1
2	Control suspension	16.53	23.74
3	Pectin 5 mg/L	23.65	32.61
4	Pectin 10 mg/L	21.33	31.59
5	Pectin 15 mg/L	18.62	27.8
6	MnCl_2_ 5 mg/L	18.84	34.36
7	MnCl_2_ 10 mg/L	16.44	23.46
8	MnCl_2_ 15 mg/L	25.58	37.23
9	Thymoquinone	0.363	12.83
10	Thymol	9.51	15.33
11	Ascorbic acid	13.66	—
12	FeSO_4_	—	19.02

**Table 4:** Minimum inhibitory concentration of *N. sativa* suspension cultures under pectin and MnCl_2_ elicitation.

Pathogenic bacterial strains	Minimum inhibitory concentration (*µ*g mL^−1^) of different extracts							
	A	B	C	D	E	F	G	H
*E. coli *	3.61 ± 0.5	2.35 ± 0.8	3.20 ± 0.6	2.90 ± 0.3	2.46 ± 0.5	3.25 ± 0.3	2.46 ± 0.2	4.15 ± 0.3
*S. typhi *	3.90 ± 0.4	2.65 ± 0.3	3.85 ± 0.5	3.56 ± 0.4	3.10 ± 0.3	3.75 ± 0.4	2.75 ± 0.4	4.86 ± 0.3
*B. cereus *	4.02 ± 0.2	2.40 ± 0.3	3.80 ± 0.2	3.15 ± 0.2	2.55 ± 0.6	3.36 ± 0.3	2.49 ± 0.3	4.55 ± 0.4
*S. aureus *	4.15 ± 0.5	2.60 ± 0.5	3.65 ± 0.7	3.20 ± 0.3	2.58 ± 0.6	3.69 ± 0.7	2.54 ± 0.5	4.69 ± 0.7
*K. pneumoniae *	4.20 ± 0.6	2.55 ± 0.4	3.32 ± 0.4	3.65 ± 0.4	2.65 ± 0.3	3.95 ± 0.2	2.59 ± 0.2	5.05 ± 0.2

A = epicotyl extract; B = control suspension culture; C = pectin 5 mg/L elicitation; D = pectin 10 mg/L elicitation; E = pectin 15 mg/L elicitation; F = MnCl_2_ 5 mg/L elicitation; G = MnCl_2_ 10 mg/L elicitation; H = MnCl_2_ 15 mg/L elicitation; “−” = absent; and “+” = present.

**Table 5:** Zone of inhibition of *N. sativa* suspension cultures under pectin and MnCl_2_ elicitation.

S. number	Organism	Diameter of zone of inhibition (mm)												
		A	B	C	D	E	F	G	H	Antibiotics				
										ST	CF	DO	AM	OF
1	*E. coli *	22 ± 0.3	32 ± 0.4	22 ± 0.3	24 ± 0.4	27 ± 0.5	20 ± 0.2	30 ± 0.2	18 ± 0.2	12 ± 0.2	12 ± 0.4	16 ± 0.4	11 ± 0.3	10 ± 0.2
2	*S. typhi *	21 ± 0.6	28 ± 0.4	18 ± 0.3	20 ± 0.3	20 ± 0.4	18 ± 0.6	28 ± 0.4	16 ± 0.4	16 ± 0.3	11 ± 0.3	0	0	0
3	*B. cereus *	20 ± 0.7	32 ± 0.6	18 ± 0.4	22 ± 0.3	30 ± 0.7	20 ± 0.3	28 ± 0.2	17 ± 0.8	14 ± 0.2	18 ± 0.4	12 ± 0.2	12 ± 0.4	13 ± 0.4
4	*S. aureus *	20 ± 0.3	30 ± 0.2	20 ± 0.2	21 ± 0.7	26 ± 0.3	19 ± 0.3	30 ± 0.6	17 ± 0.3	19 ± 0.5	14 ± 0.5	10 ± 0.2	0	0
5	*K. pneumoniae *	18 ± 0.2	30 ± 0.5	21 ± 0.2	20 ± 0.6	24 ± 0.3	17 ± 0.8	28 ± 0.4	15 ± 0.7	20 ± 0.3	14 ± 0.2	13 ± 0.4	0	15 ± 0.5

A = epicotyl extract; B = control suspension culture; C = pectin 5 mg/L elicitation; D = pectin 10 mg/L elicitation; E = pectin 15 mg/L elicitation; F = MnCl_2_ 5 mg/L elicitation; G = MnCl_2_ 10 mg/L elicitation; H = MnCl_2_ 15 mg/L elicitation; “−” = absent and “+” = present; ST = streptomycin; CF = ciprofloxacin; DO = doxycycline; AM = ampicillin; OF = ofloxacin; and susp. = suspension.
